# Site-selective ^1^H/^2^H labeling enables artifact-free ^1^H CPMG relaxation dispersion experiments in aromatic side chains

**DOI:** 10.1007/s10858-019-00275-z

**Published:** 2019-09-10

**Authors:** Heiner N. Raum, Julia Schörghuber, Matthias Dreydoppel, Roman J. Lichtenecker, Ulrich Weininger

**Affiliations:** 1 Institute of Physics, Biophysics, Martin-Luther-University Halle-Wittenberg, 06120 Halle, Germany; 2 Institute of Organic Chemistry, University of Vienna, 1090 Vienna, Austria

**Keywords:** Conformational exchange, Protein dynamics, Aromatic side chains, Strong couplings

## Abstract

Aromatic side chains are often key residues in enzyme active sites and protein binding sites, making them attractive probes of protein dynamics on the millisecond timescale. Such dynamic processes can be studied by aromatic ^13^C or ^1^H CPMG relaxation dispersion experiments. Aromatic ^1^H CPMG relaxation dispersion experiments in phenylalanine, tyrosine and the six-ring moiety of tryptophan, however, are affected by ^3^J ^1^H–^1^H couplings which are causing anomalous relaxation dispersion profiles. Here we show that this problem can be addressed by site-selective ^1^H/^2^H labeling of the aromatic side chains and that artifact-free relaxation dispersion profiles can be acquired. The method has been further validated by measuring folding–unfolding kinetics of the small protein GB1. The determined rate constants and populations agree well with previous results from ^13^C CPMG relaxation dispersion experiments. Furthermore, the CPMG-derived chemical shift differences between the folded and unfolded states are in excellent agreement with those obtained directly from the spectra. In summary, site-selective ^1^H/^2^H labeling enables artifact-free aromatic ^1^H CPMG relaxation dispersion experiments in phenylalanine and the six-ring moiety of tryptophan, thereby extending the available methods for studying millisecond dynamics in aromatic protein side chains.

## Introduction

Aromatic amino acids are an important subgroup of amino acids. They are bulky and responsible for a significant proportion of the protein hydrophobic core, where they typically form pairs or clusters making specific aromatic–aromatic interactions (Burley and Petsko 1985, 1989). They are overrepresented in protein binding interfaces where they contribute significantly to the binding free energy (Birtalan et al. 2010; Bogan and Thorn 1998; Lo Conte et al. 1999) and, in the form of His and Tyr, are key residues in enzyme catalysis (Bartlett et al. 2002). Even more, Phe and Tyr undergo frequent 180° rotations of the χ_2_ angle (‘ring flips’) and thereby provide unique information of transient ‘breathing’ processes of proteins (Li et al. 1999; Wagner 1980; Wagner et al. 1976; Weininger et al. 2014b). All of these reasons make aromatic side chains interesting and useful probes for studying protein dynamics on the millisecond time scale.

Conformational transitions on this time scale are often linked to biological functions (Mittermaier and Kay 2009) and transiently populated high-energy states play important roles in enzyme catalysis (Boehr et al. 2006; Cole and Loria 2002; Eisenmesser et al. 2002) or ligand binding (Demers and Mittermaier 2009; Malmendal et al. 1999). Such transitions between different conformations generally lead to a modulation of NMR parameters as the chemical shift (Gutowsky and Saika 1953) or residual dipolar couplings (Igumenova et al. 2007; Vallurupalli et al. 2007), resulting in exchange contributions to transverse relaxation rate constants. This can be probed by NMR relaxation dispersion methods from which one can gain unique information on the structures, thermodynamics and kinetics of the underlying processes (Palmer 2004; Palmer et al. 2001).

Protein dynamics on the millisecond time scale in aromatic side chains can be studied by ^13^C (Weininger et al. 2012) and ^1^H (Raum et al. 2018) CPMG relaxation dispersion experiments. The key requirement for ^13^C relaxation dispersion experiments is site-selective ^13^C labeling of aromatic side chains (Lundström et al. 2012; Schörghuber et al. 2018; Weininger 2019), which eliminates ^1^J ^13^C–^13^C couplings. To date, there are several well established labeling strategies, that achieve this goal (Kasinath et al. 2013; Lichtenecker 2014; Lichtenecker et al. 2013; Lundström et al. 2007; Milbradt et al. 2015; Schörghuber et al. 2015, 2017a, b; Teilum et al. 2006; Weininger 2017a, b). ^13^C relaxation dispersion experiments for the study of dynamics on the ms (Weininger et al. 2012) and µs (Weininger et al. 2014a) time scale have been developed and applied on the characterization of ring flips (Weininger et al. 2013, 2014b) and transient histidine tautomerization (Weininger et al. 2017). In contrast, sole site-selective ^13^C labeling is not sufficient to enable artifact-free ^1^H CPMG relaxation dispersion experiments. It eliminates artifacts from possible strong ^13^C–^13^C couplings, but sizeable ^3^J ^1^H–^1^H couplings (that exist in Phe, Tyr, and the 6-ring moiety of Trp) cause severe artifacts (Raum et al. 2018). Therefore, additional site-selective ^1^H/^2^H labeling is required.

Here we demonstrate that in site-selective ^1^H/^2^H labeled aromatic side chains the artifact caused by ^3^J ^1^H–^1^H couplings is gone and flat relaxation dispersion profiles can be measured in the absence of exchange. Artifact-free relaxation dispersion profiles can be acquired if chemical exchange is present, and meaningful (exchange rates and populations) and correct (chemical shift differences) parameters of this process can be determined. This has been demonstrated on the folding/unfolding of protein GB1 at high temperatures.

## Materials and methods

### Protein samples

Uniformly ^1^H and site-selective ^13^C labeled, using 2 g/L 2-^13^C_1_ glucose (Lundström et al. 2007), as well as site-selective ^1^H and ^13^C labeled, using specific synthesized precursors (Lichtenecker 2014; Lichtenecker et al. 2013; Schörghuber et al. 2015) (80 mg/L for Phe and Tyr, 10 mg/L for Trp), GB1 (QDD variant) was expressed and purified as described in (Lindman et al. 2006). Samples contained 990 µM (uniformly ^1^H labeled) or 440 µM (site-selective ^1^H labeled) protein in 20 mM HEPES and 10% (v/v) D_2_O at pH 7.0. Small amounts of NaN_3_ were added.

### NMR spectroscopy

All experiments were acquired on a Bruker Avance III spectrometer at a static magnetic field of 14.1 T or 18.8 T and 298 K or 313 K. ^1^H CPMG relaxation dispersion experiments were performed using a relaxation compensated approach as published before (Raum et al. 2018). A constant relaxation period of 20 ms was chosen in all experiments, except for 18.8 T and 313 K where 40 ms was chosen. Refocusing frequencies are between 100 (50 for 18.8 T) and 1000 Hz and B1 field strengths for the CPMG pulses are 16 kHz (14.1 T) and 18.5 kHz (18.8 T). During the CPMG period the ^1^H carrier was put in the aromatic region (6.7 ppm). No ^2^H decoupling was used for the experiments. Experiments were typically performed with 72 number of scans resulting in a experimental time of 2 days. This was done to assure the highest data quality possible for the establishment of the method. Further the dispersion step is quite moderate and there are only three positions for the global fit. Spectra were processed with NMRPipe (Delaglio et al. 1995) and analyzed with PINT (Ahlner et al. 2013) or NMRView (Johnson 2004). The spectra have not been referenced to DSS.

### Data analysis

Measurement uncertainties of relaxation rates were estimated as the average standard deviation of double measurements. CPMG relaxation dispersion experiments were fitted globally to the Carver-Richards equation (Carver and Richards 1972; Davis et al. 1994). Data modeling utilized the Levenberg–Marquardt (Press et al. 2002) nonlinear least-squares optimization algorithm implemented in MATLAB. For error estimation, Monte-Carlo simulations with 1000 steps were executed. Derived Δδ values were compared with ^1^H shift differences between native and unfolded signals at 40 °C.

## Results and discussion

GB1 is a small, 56 residue protein containing 6 aromatic amino acids: 1 Trp (W43), 2 Phe (F30 and F52) and 3 Tyr (Y3 and Y33 and Y45).

### Site-selective ^1^H/^2^H labeling in the aromatic side chains of GB1

**Fig. 1:**
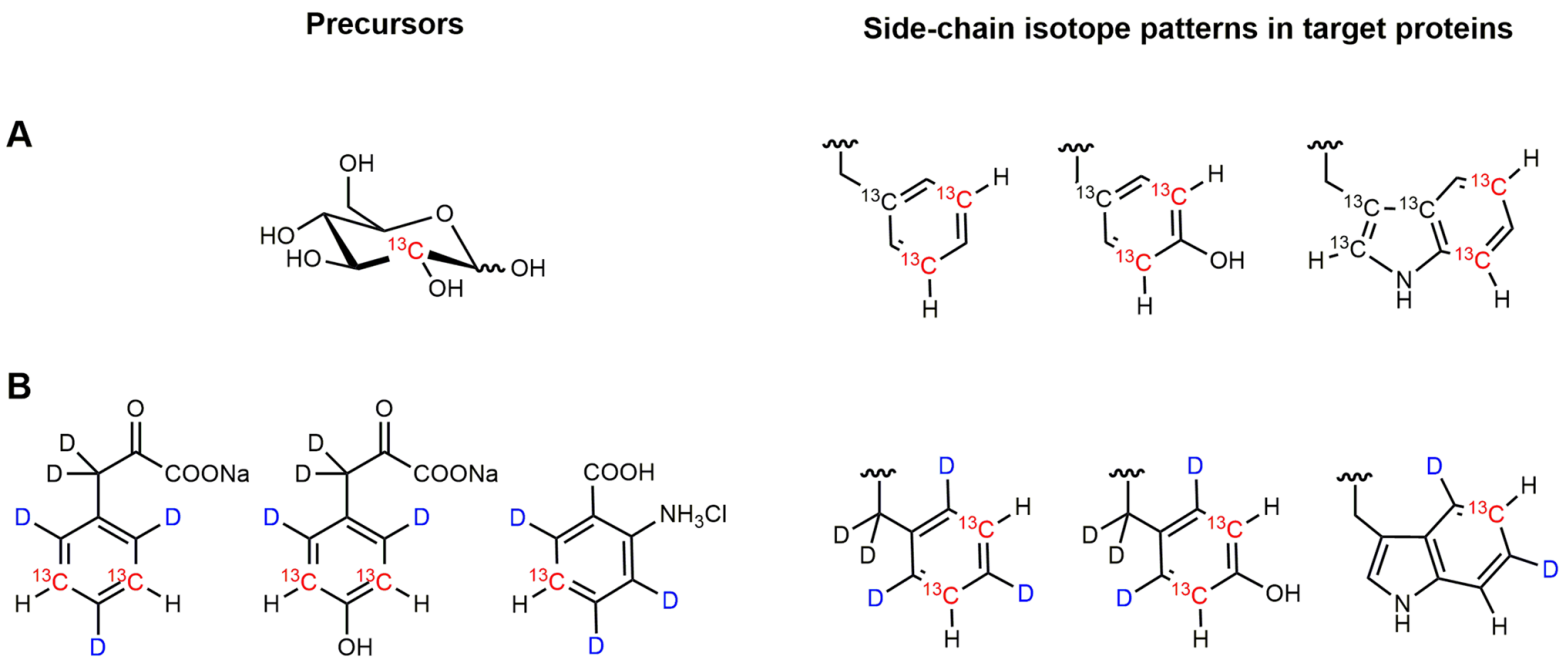
Aromatic side chains (Trp, Phe and Tyr) with ^1^H/^2^H and ^13^C labeling resulting from 2-^13^C_1_ glucose (**a**) and specific synthesized precursors (**b**). ^13^C with an attached proton are colored red. ^2^H in the aromatic ring is colored blue. ^13^C incorporation from glucose is 22% (Fε*), 19% (Yε*), 24% (Wζ3) and 12% (Wζ2). ^13^C incorporation (together with ^2^H incorporation at adjacent positions) from the specific precursors is 100% (Fε*) and > 75% (Wζ3). The same should be expected for Yε* (but did not in this attempt)

Both labeling methods result in site-selective ^13^C labeling in Phe ε* (F30 and F52), Tyr ε* (Y3 and Y33 and Y45) and Trp ζ3 (W43). In the uniformly ^1^H labeled sample (based on 2-^13^C_1_ glucose) Trp δ1 and ζ2 (Fig. 1) are labeled additionally. The resulting spectra are highly comparable, signals from the site-selective ^1^H labeled sample showed reduced linewidths in ^1^H (about 25% reduced). If normalized by the protein concentration the site-selective ^1^H labeled sample will display 5.5 times the signal strength for Phe ε* (F30 and F52) and three times the signal strength for Trp ζ3 (W43). In case of Phe ε*, with about 20% ^13^C incorporation from 2-^13^C_1_ glucose (Weininger 2017a), this translates to an apparent 110% ^13^C incorporation in the site-selective ^1^H labeled sample. This can be explained by a close to 100% ^13^C incorporation, 5% gain because losses from ^3^J ^1^H–^1^H couplings during the INEPT transfer periods are suppressed and additional gain from improved ^1^H relaxation. In case of Trp ζ3, with about 25% ^13^C incorporation from 2-^13^C_1_ glucose (Weininger 2017a) this translates to an apparent 75% ^13^C incorporation in the site-selective ^1^H labeled sample. Applying the same reasoning for Trp ζ3, one can estimate around 70% incorporation in case of the site-selective ^1^H labeled sample (at 10 mg/L precursor). In case of Tyr the site-selective labeling did not work well (around 4% ^13^C incorporation). The reason for this is not clear. As a direct consequence of this only a limited number of experiments could be performed on Tyr. On the other hand this highlights the selective incorporation of the Phe precursor in Phe. Results of ^13^C incorporation are summarized in Table 1.

**Table 1:** Site-selective ^13^C incorporation (in %) in aromatic side-chains using glucose (Glc) or synthesized precursors (SP)

	2-^13^C Glc^a^	SP Phe^b^	SP Tyr^b^	SP Trp^b^
Phe γ	55	1	1	1
Phe δ*	4	1	1	1
Phe ε*	22	99	1	1
Phe ζ	1	1	1	1
Tyr γ	n.d.	1	1	1
Tyr δ*	4	1	1	1
Tyr ε*	19	1	?^c^	1
Tyr ζ	0	1	1	1
Trp γ	10	1	1	1
Trp δ1	49	1	1	1
Trp δ2	n.d.	1	1	1
Trp ε2	n.d.	1	1	1
Trp ε3	2	1	1	1
Trp ζ3	24	1	1	70
Trp η2	2	1	1	1
Trp ζ2	12	1	1	1

^a^From (Weininger 2017a). ^13^C incorporation in direct neighborhood of the positions of interest (Phe ε*, Tyr ε*, Trp ζ3, Trp ζ2) is 1%. Higher values arise from scrambling in molecules that are not ^13^C labeled at the positions of interest

^b^The synthesized precursors label exclusively one amino acid

^c^Incorporation at Tyr ε* is expected to be 99% (analogue to Phe) but only was 4% in this work

**Fig. 2:**
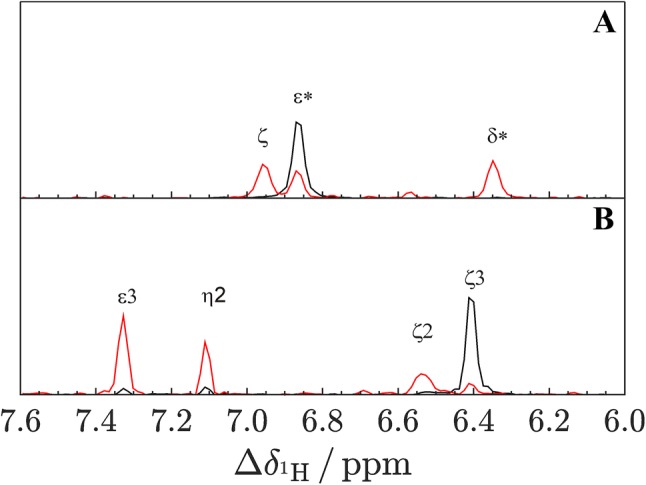
1D ^1^H (constant ^13^C chemical shift) slices from ^1^H^13^C HSQC-TOCSY spectra from uniformly ^1^H labeled (red) and site-selective labeled (black) GB1. The spectra at the top (**a**) are from the slice along the ^13^C chemical shift of F30Cε* (128.21 ppm) and the spectra at the bottom (**b**) are from the slice along the ^13^C chemical shift of W43Cζ3 (118.15 ppm). Each spectrum is normalized to the maximum intensity of the corresponding peak of a ^1^H^13^C HSQC spectrum measured under same conditions. Signals are labeled. Ratios of auto to (vicinal) cross signals in the uniformly ^1^H labeled cases are 27/73 (**a**) and 8/92 (**b**), in the site-selective ^1^H cases 100/0 (**a**) and 90/10 (**b**)

In order to evaluate the amount of vicinal protons (^3^J) to the ^13^C bound proton we performed 2D ^1^H^13^C-HSQC-^1^H^1^H-TOCSY experiments. For the uniformly ^1^H labeled samples we observe strong cross signals to vicinal (and for Trp ζ3 ^4^J) protons (Fig. 2, red). These are completely absent (Phe ε*) or strongly reduced (Trp ζ3) in the site-selective ^1^H labeled sample, indicating complete (> 98%, Phe ε*) or a high amount of (about 90%, Trp ζ3) deuteration of vicinal protons, that are believed to cause artifacts in aromatic ^1^H CPMG relaxation dispersion experiments (Raum et al. 2018). In summary, labeling by synthetic ^1^H and ^13^C selective precursors can be seen as perfect (Phe ε*) or pretty good (Trp ζ3) in terms of producing high amounts of isolated ^1^H–^13^C spin pairs in aromatic side chains. Labeling for Tyr ε* are shown to be on the same level in previous attempts (Lichtenecker et al. 2013), but did not work properly in this work.

### Artifact-free ^1^H CPMG relaxation dispersion profiles by site-selective ^1^H/^2^H labeling

**Fig. 3:**
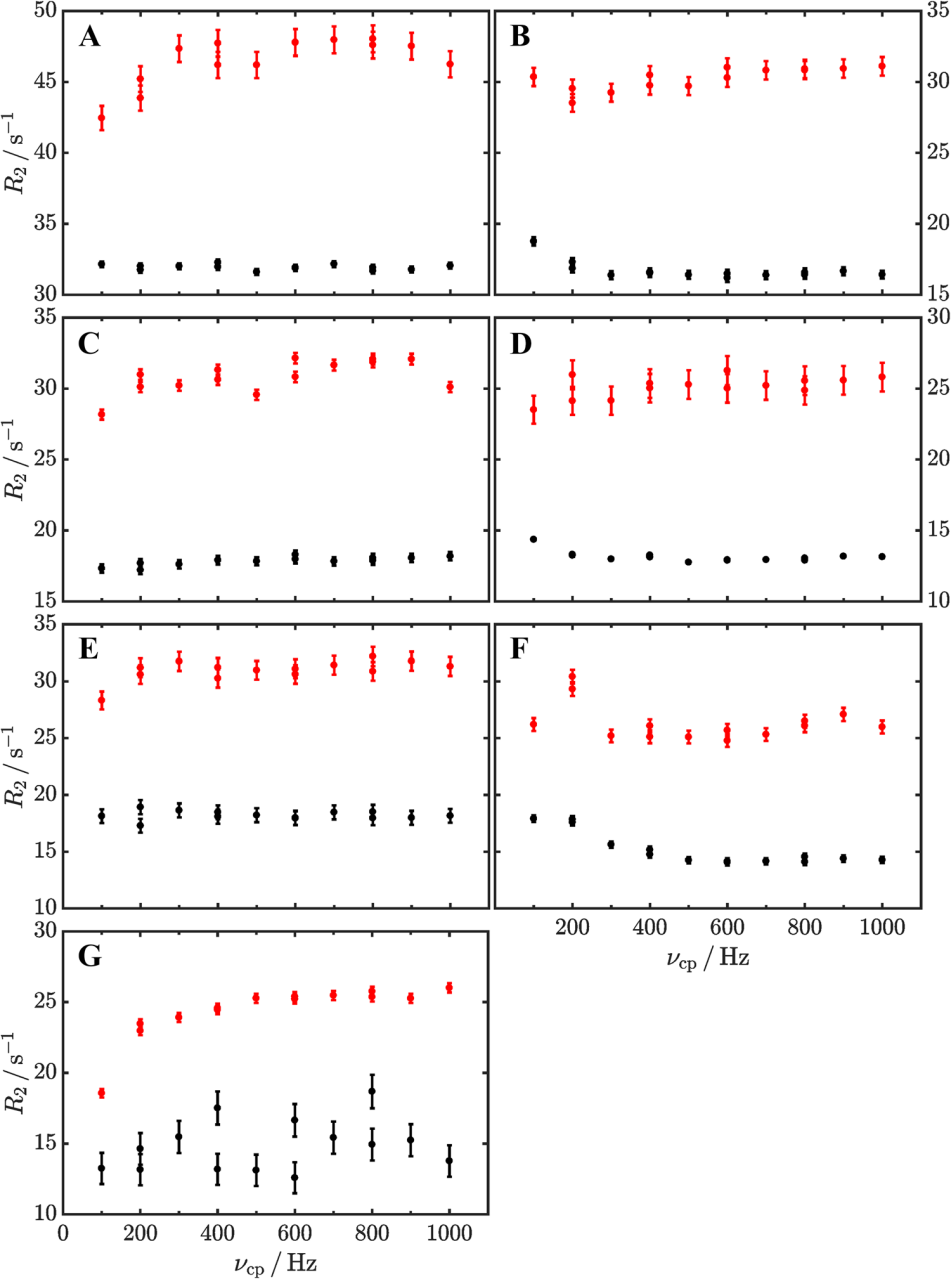
Aromatic ^1^H CPMG relaxation dispersion profiles of uniformly ^1^H labeled (colored in red) and site-selective labeled (black) GB1 at 298 K (**a**, **c**, **e** and **g**, no exchange) and 313 K (**b**, **d** and **f**, exchange) at a static magnetic field of 14.1 T. For F30ε* (**a**), F52ε* (**c**) and W43ζ3 (**e**) flat relaxation dispersion profiles are obtained with the site-selective labeled sample at 298 K and unperturbed relaxation dispersion profiles are obtained at 313 K (**b**, **d**, **f**). The uniformly labeled sample shows artificial profiles affected by ^3^J ^1^H–^1^H couplings in all cases. F30ε* at 298 K is additionally affected by ring flips that cause a constant increase in *R*_2_ values. For Y33ε* (**g**), the artifact is significantly reduced with the site-selective labeled sample

Next, we recorded ^1^H CPMG relaxation profiles on both GB1 samples at 25 °C (no exchange conditions) and 40 °C (exchange conditions) (Weininger 2019). At 25 °C, we observe anomalous relaxation dispersion profiles for the uniformly ^1^H labeled sample that are caused by ^3^J ^1^H–^1^H couplings (Fig. 3a, c, e, red). In contrast, we observe flat relaxation dispersion profiles for the site-selective labeled sample (Fig. 3a, c, e, black), as one would expect for conditions with no chemical exchange. Resulting RMSD values for the flat dispersions are: 0.18 s^−1^ (F30), 0.29 s^−1^ (F52) and 0.39 s^−1^ (W43). Furthermore, *R*_2_ values at high refocusing frequencies are around 15 s^−1^ higher in the uniformly ^1^H labeled sample. 8 s^−1^ can be directly attributed to the ^3^J ^1^H–^1^H coupling (Raum et al. 2018), the rest can be interpreted as improved ^1^H relaxation in the site-selective ^1^H labeled sample. In case of Tyr (Fig. 3g), the observation was the same. Because of the low labeling yield in case of site-selective ^1^H/^2^H labeling, the recorded relaxation dispersion profile (Fig. 3e, black) is noisier. Additionally, since contribution from uniformly ^1^H labeled protein at ^13^C natural abundance are comparable to the site-selective ^1^H/^2^H labeled protein (1–4%), the artifact is reduced but still there. Under exchange conditions at 40 °C one can record pronounced relaxation dispersion profiles for the site-selective labeled sample (Fig. 3b, d, f, black). In contrast, relaxation dispersion profiles appear to be flat (Fig. 3b, red) or heavily perturbed (Fig. 3d, f, red), for the uniformly ^1^H labeled samples. This can be visualized as a superposition of the artifact caused by the ^3^J ^1^H–^1^H couplings and the relaxation dispersion profiles caused by chemical exchange. In summary, we have confirmed earlier findings, that ^3^J ^1^H–^1^H couplings are causing artifacts in aromatic ^1^H CPMG relaxation profiles (if they are larger than 2 Hz), and established that this problem can be eliminated by the use of site-selective ^1^H labeled precursors.

### Unfolding of GB1 by ^1^H CPMG relaxation dispersion

**Fig. 4:**
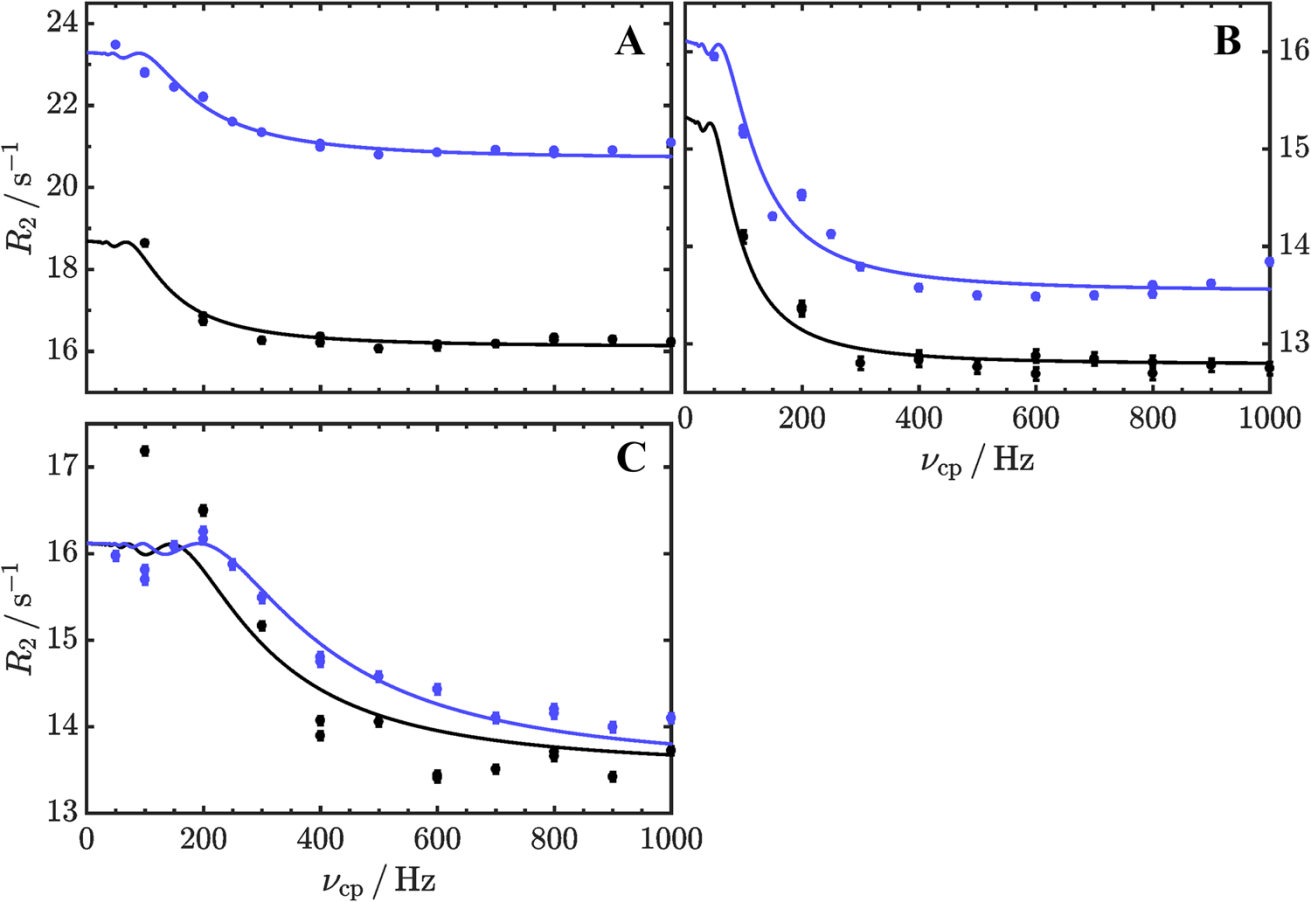
Aromatic ^1^H CPMG relaxation dispersion profiles acquired on a ^1^H selective-labeled 440 µM sample of GB1 in 20 mM HEPES, pH 7, at 40 °C and static magnetic field strengths of 14.1 (black) and 18.8 (blue). Data are shown for F30ε* (**a**), F52ε* (**b**) and W43ζ3 (**c**). Solid lines represent the global fit of a two-state exchange model to the experimental data. The resulting exchange rate *k*_ex_ is (94 ± 5) s^−1^, the population of the unfolded state *p*_u_ (2.8 ± 0.1)%. Differences in chemical ^1^H shift were estimated to (0.22 ± 0.01) ppm for F30ε*, (0.14 ± 0.01) ppm for F52ε* and (0.48 ± 0.01) ppm for W43ζ3 and are in good agreement with ^1^H shift differences between native and unfolded signals in the spectra, which are 0.2 ppm, 0.135 ppm and 0.475 ppm

**Fig. 5:**
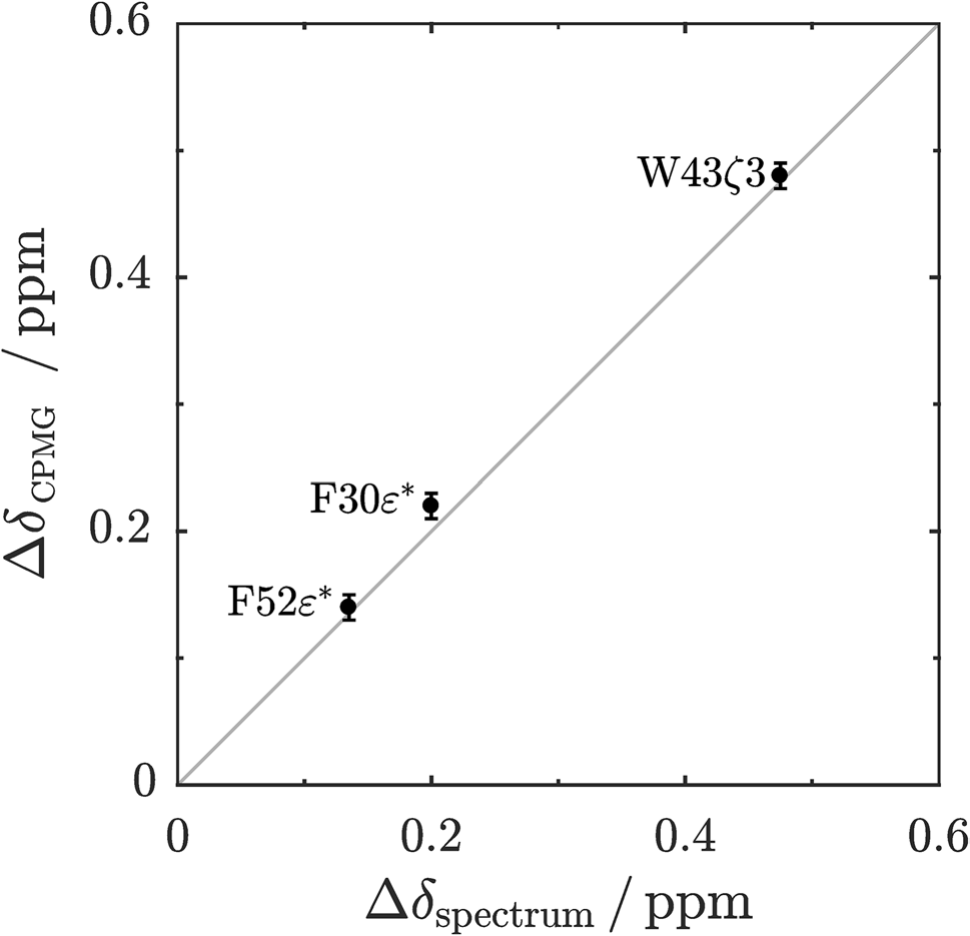
Correlation of ^1^H chemical shift differences between the folded and unfolded states of GB1 derived from CPMG relaxation dispersion experiments under native conditions and measured directly from an aromatic ^1^H–^13^C HSQC spectrum. The solid grey line represents the ideal correlation. Points are labeled. Pairwise RMSD of the points is 0.012 ppm

Finally, we applied aromatic ^1^H CPMG relaxation dispersion experiments using site-selective ^1^H labeled samples on a known exchanging system, GB1 at 40 °C (Weininger 2019). Relaxation dispersion profiles obtained at two magnetic field strengths can be fitted globally (for F30, F52 and W43) to a two-state exchange model (Fig. 4), resulting in an exchange rate constant (*k*_ex_) of (94 ± 5) s^−1^ and a population of the unfolded state (*p*_u_) of (2.8 ± 0.1)%. The derived chemical shift differences from the relaxation dispersion profiles are in excellent agreement with shift differences directly derived from spectra at 40 °C, that show the characteristics of an unfolded protein (Fig. 5). The population of the unfolded state is somewhat lower than for ^13^C CPMG relaxation dispersion experiments (Weininger 2019). These have, however, been measured at an older sample containing large amounts of salt, and salt is known to destabilize GB1 (Lindman et al. 2006). The derived populations at low salt concentration in this work are in good agreement to unfolding transitions under these conditions.

## Conclusions

We have demonstrated that artifact-free aromatic ^1^H CPMG relaxation dispersion profiles can be obtained using site-selective ^1^H labeled precursors, which produce isolated ^1^H–^13^C spin pairs in aromatic side chains. In contrast, relaxation dispersion profiles in a uniformly ^1^H labeled samples are heavily perturbed by high (7–8 Hz) ^3^J ^1^H–^1^H coupling constants. Correct parameters of unfolding of GB1 could be derived by ^1^H CPMG relaxation dispersion experiments on a site-selective ^1^H and ^13^C labeled sample. By site-selective ^1^H/^2^H labeling one can therefore extend the positions suitable for aromatic ^1^H CPMG relaxation dispersion experiments to Fε and Wζ3 specifically and Yε in principal (this work) and Fδ, Fζ, Yδ, Wε3, Wη2 and Wζ2 (using suitable precursors), thereby extending the arsenal of aromatic probes for the study of ms dynamics.
